# Robotic Cholecystectomy: Outcomes, Safety, and Value in the Era of Advanced Technology

**DOI:** 10.26502/jsr.10020518

**Published:** 2026-09-07

**Authors:** Shaanali Mukadam, Madeleine T Dang, Chang Kon Kim, Timothy J Chu, Daniel H Choi, Devendra K Agrawal

**Affiliations:** Department of Translational Research, College of Osteopathic Medicine of the Pacific, Western University of Health Sciences, Pomona, California 91766 USA

**Keywords:** Bile duct injury, Conversion to open surgery, Cost-effectiveness, Difficult gallbladder, Fluorescence cholangiography, Laparoscopic cholecystectomy, Minimally invasive surgery, Robotic cholecystectomy, Robotic surgery, Surgical outcomes

## Abstract

Robotic cholecystectomy has been increasingly adopted across general surgery, driven by potential advantages in visualization, instrument articulation, ergonomics, and fluorescence-guided biliary identification. However, its clinical value relative to conventional laparoscopic cholecystectomy remains debated, particularly given higher procedural costs and mixed comparative safety data. This narrative review synthesizes contemporary evidence from 2020 to 2025 comparing robotic and laparoscopic cholecystectomy, with emphasis on operative outcomes, perioperative recovery, complex biliary disease, cost, and patient-reported outcomes. Current data suggest that robotic cholecystectomy is associated with modestly longer operative time during early adoption, although efficiency may improve with increasing procedural familiarity and standardized workflow. Conversion to open surgery is a recurrent favorable outcome reported with robotics, particularly in difficult gallbladders, obesity, acute inflammation, and reoperative or post-inflammatory settings. Intraoperative safety findings remain heterogeneous. Population-level analyses have raised concern regarding bile duct injury, whereas contemporary institutional, registry, and nonelective analyses report low or comparable bile duct injury rates. Postoperative recovery, length of stay, wound complications, readmission, quality of life, and patient satisfaction are generally similar between approaches, with limited evidence of modest early pain or recovery advantages in selected studies. Economic data are more consistent, showing higher direct and indirect costs for robotic cholecystectomy and unfavorable cost-effectiveness in routine elective disease. Overall, contemporary evidence does not support routine replacement of laparoscopic cholecystectomy with robotic cholecystectomy for uncomplicated gallbladder disease. The robotic platform may be best positioned for selective use in technically complex cases where enhanced visualization, articulation, and operative control could reduce conversion risk or facilitate minimally invasive completion. Future prospective, severity-adjusted studies incorporating surgeon experience, patient-centered recovery, and full cost accounting are needed to clarify where robotic cholecystectomy provides measurable clinical value.

## Introduction

Cholecystectomy has evolved from an open operation to a predominantly minimally invasive procedure, with laparoscopic cholecystectomy becoming the standard approach for gallbladder removal over the past three decades [1]. More recently, continued innovation in minimally invasive surgery has expanded the use of robotic platforms across general surgery. Use of robotic surgery in common general procedures increased substantially between 2012 and 2018 [2–7], and robotic cholecystectomy has similarly expanded in clinical practice, with national data demonstrating marked growth in utilization in recent years [8]. This expansion has been driven in part by interest in potential technical advantages of the robotic platform, including enhanced dexterity, three-dimensional visualization, and improved ergonomics. In challenging cases, such as difficult gallbladders with severe inflammation or adhesions, these features may offer greater precision and stability and may help reduce conversion to open surgery [9,10].

Despite this increasing adoption, the clinical role of robotic cholecystectomy remains unsettled. Some analyses have raised concern regarding safety and questioned whether the routine use of robotics is justified in the absence of clear offsetting benefit [6]. In contrast, other contemporary studies have suggested that robotic cholecystectomy may be associated with lower rates of selected adverse outcomes and conversion to open surgery compared with laparoscopy [12], while institutional data have also suggested shorter hospital stay and lower conversion rates in elective cases, even among patients with greater comorbidity [13]. However, systematic reviews and meta-analyses have not demonstrated consistent clinical superiority of robotic cholecystectomy in routine postoperative outcomes [14]. Overall, current evidence suggests that robotic cholecystectomy may offer advantages in selected settings, but consistent improvement in routine perioperative outcomes has not been established.

Economic considerations have further intensified this debate. Recent studies have shown that robotic cholecystectomy is associated with higher direct procedural costs than laparoscopic cholecystectomy, often without major differences in length of stay, complications, or reintervention [15]. Meta-analytic data similarly indicate that laparoscopy remains the more cost-effective approach in routine practice [9]. In a high-volume procedure such as cholecystectomy, these differences have important implications for the value-based adoption of robotic technology.

Given the rapid adoption of robotic cholecystectomy alongside ongoing debate regarding its safety, value, and clinical role, a contemporary synthesis of the evidence is warranted. This review examines recent evidence comparing operative outcomes, perioperative recovery, and economic implications of robotic and laparoscopic cholecystectomy.

## Literature Search Methodology

**Figure 1:**
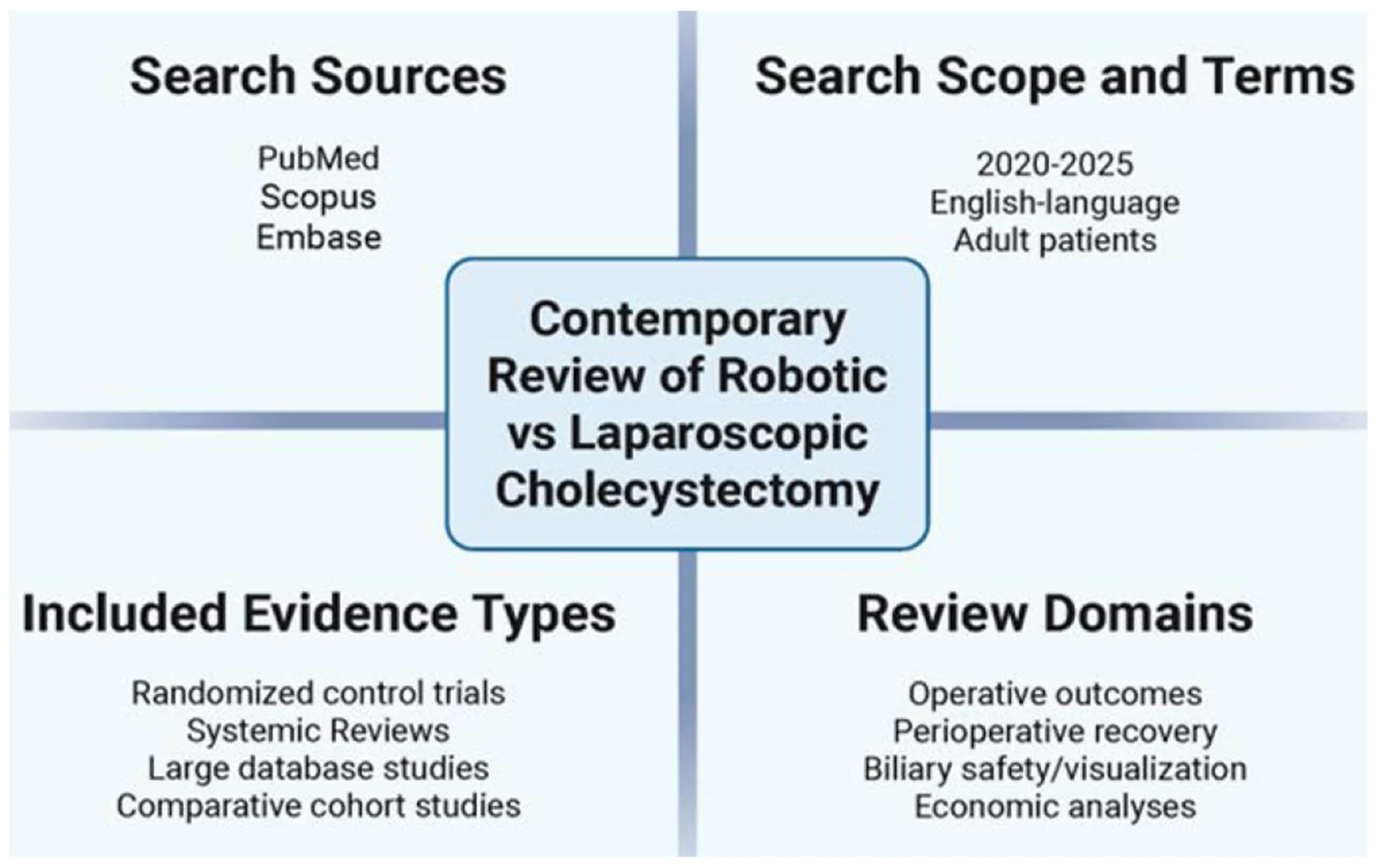
An overview of the search methodology on the contemporary review of robotic vs laparoscopic cholecystectomy.

A targeted literature search was conducted using PubMed, Scopus, and Embase to identify studies published between 2020 and 2025 comparing robotic and laparoscopic cholecystectomy in adult patients. Earlier studies were included when they provided foundational or directly relevant comparative data on ergonomics, patient-reported outcomes, cost, or surgical volume. Search terms included combinations of “robotic cholecystectomy”, “laparoscopic cholecystectomy”, “robotic-assisted cholecystectomy”, “bile duct injury”, “conversion to open surgery”, and “cost-effectiveness”. Emphasis was placed on studies reporting operative outcomes, perioperative recovery, and economic analyses. Eligible study designs included randomized control trials, systematic reviews, large database analyses, and comparative cohort studies (Figure 1).

## Operative and Perioperative Outcomes

### Operative Time and Setup

**Figure 2:**
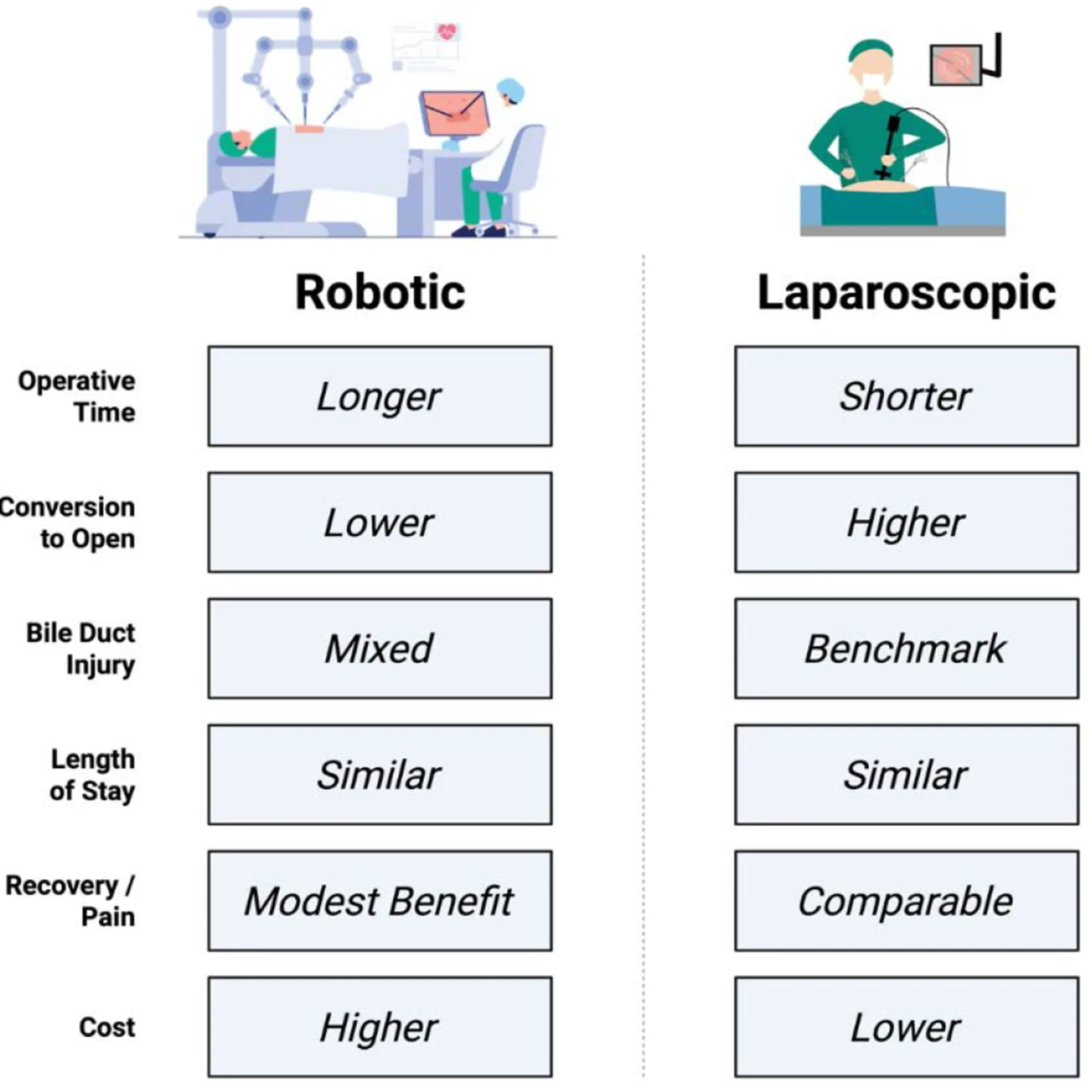
Comparison of various factors between the robotic and laparoscopic procedures. Figure created by the authors. Header illustrations adapted from publicly available online images: robotic surgery illustration from IconScout, “Robotic Surgery Illustration” by SpacePixel Creative, asset ID 12825766; laparoscopic surgery illustration retrieved from the Shukan Hospital & IVF Centre “Safety in Laparoscopy” webpage. Accessed August 16, 2026.

Robotic cholecystectomy is generally associated with slightly longer operative time than laparoscopy, largely because of docking and setup requirements. Contemporary large-scale and meta-analytic studies suggest that this difference is modest, typically on the order of 8 to 12 minutes [8,12,14]. Single-center and workflow-based studies likewise show improved operative efficiency with greater procedural familiarity and more standardized setup [16–20]. In some elective series, no meaningful operative time difference has been observed between robotic and laparoscopic cholecystectomy [21] (Figure 2).

### Conversion to Open Surgery

Robotic cholecystectomy has been associated with lower conversion rates to open surgery than laparoscopy. Large national databases show approximately half the odds of conversion with the robotic approach (adjusted OR ≈ 0.4–0.5) [8,12]. Institutional series report conversion rates<1% for robotic cases compared with 2–3% for laparoscopic procedures in comparable populations [16,18,22]. The difference may reflect improved instrument articulation and visualization, which can facilitate safer dissection in difficult anatomy (Figure 2).

## Intraoperative Complications (Bile Duct Injury, Bleeding, Perforation)

Comparative data on intraoperative complications remain mixed, particularly with respect to bile duct injury (BDI) (Figure 2). Population-level analyses have reported higher bile duct injury rates with robotic cholecystectomy than with laparoscopy, including one large Medicare analysis in which major bile duct injury occurred in 0.72% of robotic cases versus 0.23% of laparoscopic cases [16].

However, subsequent institutional and registry studies involving experienced operators have not replicated these elevated rates. Contemporary data involving over 4,000–5,000 robotic cholecystectomies report major BDI rates of 0.0–0.1%, comparable to laparoscopic outcomes [10,16,23,24]. Similarly, a 2025 systematic review and meta-analysis of 1,073,587 nonelective procedures found no significant difference in BDI rates between robotic and laparoscopic cholecystectomy [25].

Bleeding complications are uncommon with both techniques. In one multicenter series, transfusion occurred in 0.5% of robotic versus 1.3% of laparoscopic cases (p = 0.017) [14], although pooled analyses have not shown consistent differences in blood loss or transfusion requirements [26]. Gallbladder perforation and bile spillage may occur less often with robotic cholecystectomy, with data suggesting 74% lower odds than with laparoscopy [10] and institutional series reporting perforation rates of 1–2% versus 3–4%, respectively [16,18].

In aggregate, available data suggest that robotic cholecystectomy achieves intraoperative safety broadly comparable to laparoscopy in many contemporary series, although interpretation of BDI risk remains sensitive to study design, case selection, and surgeon experience [8,10,12,16,18].

## Visualization and Dissection Safety (ICG Fluorescence and Critical View)

**Figure 3:**
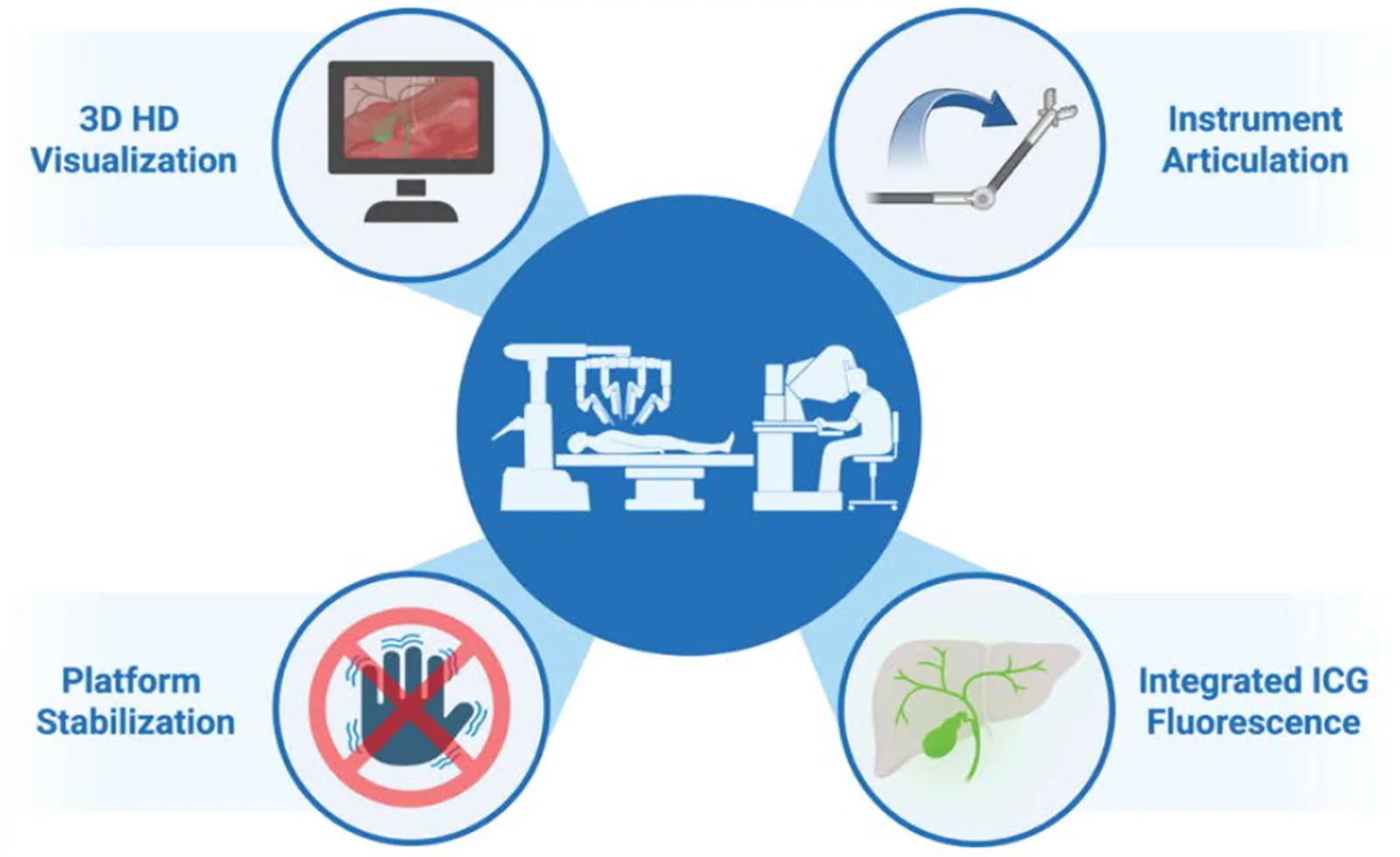
Use of various procedures and strategies from the visualization and safety point of view. Figure created by the authors. Central robotic surgery silhouette adapted from the publicly available NicePNG image “Robotic Surgery,” image ID 8809590. Accessed August 16, 2026.

High-definition 3D optics and integrated fluorescence imaging may improve anatomic identification during robotic cholecystectomy. Near-infrared fluorescence with indocyanine green facilitates visualization of biliary anatomy and, in pooled analyses, has been associated with lower conversion rates and improved duct identification [27]. However, because indocyanine green can also be used in laparoscopic surgery, these benefits should not be attributed exclusively to the robotic platform. When restricted to randomized trials, fluorescent cholangiography has improved identification of key biliary structures without demonstrating clear reductions in bile duct injury, conversion, operative time, or postoperative complications [28] (Figure 3).

Institutional series have also reported favorable operative efficiency and low biliary complication rates with routine use of fluorescence guidance, including in obese or inflamed cases [29].

Prospective robotic studies report critical-view-of-safety achievement exceeding 98% and no biliary misidentifications when ICG is used [16,18]. Fluorescence-guided imaging appears to be a useful adjunct during robotic cholecystectomy, although direct evidence linking it to improved hard clinical outcomes remains limited (Figure 3).

## Surgeon Ergonomics and Fatigue

**Figure 4:**
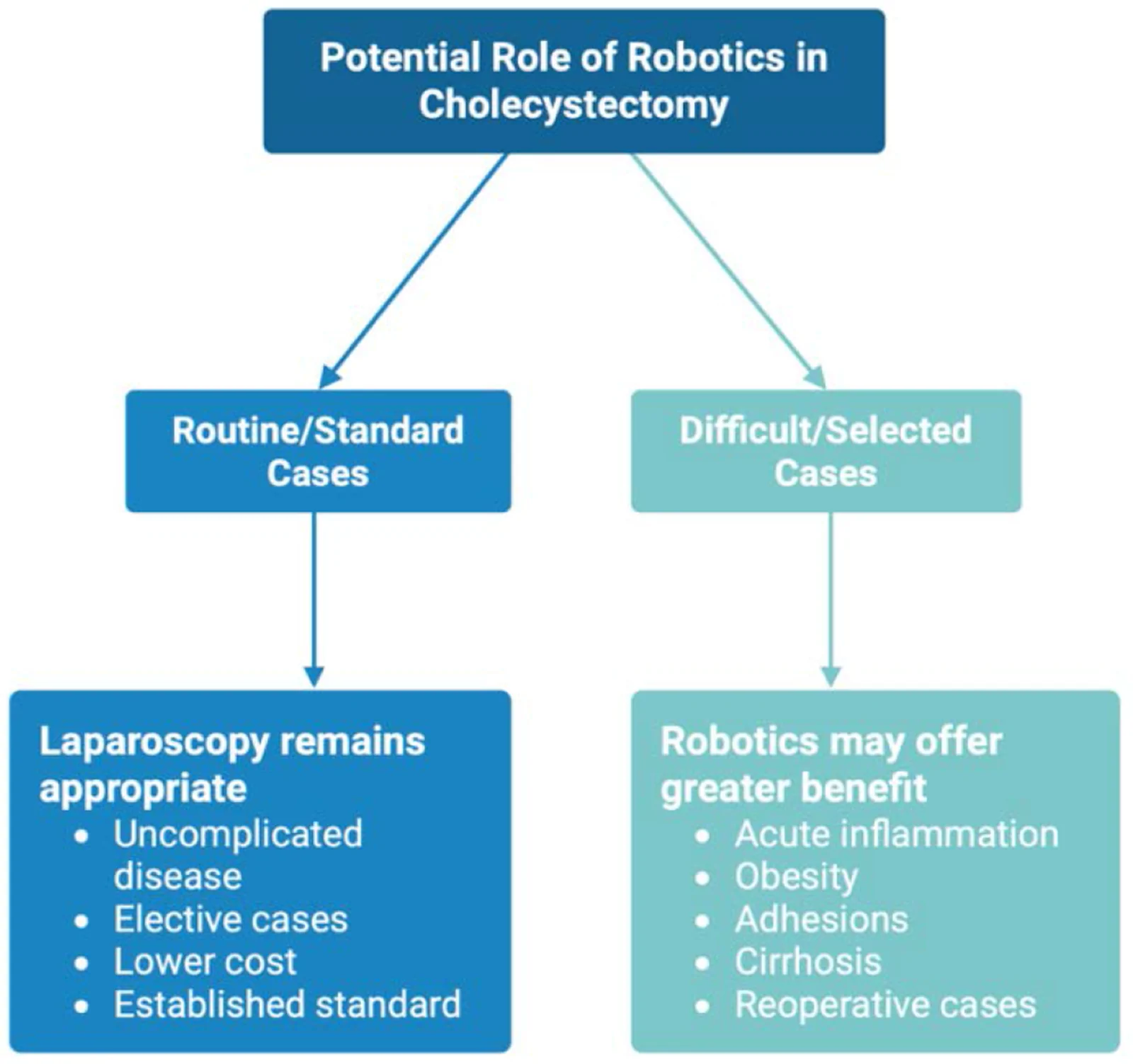
Potential role of robotics vs laparascopy in cholecystectomy in routine/standard cases vs difficult/selected case.

Robotic surgical platforms may offer ergonomic advantages over conventional laparoscopy by allowing the surgeon to operate seated with arm, wrist, and head support while controlling wristed instruments through motion scaling and tremor filtration [30]. Experimental and survey-based studies have shown lower upper-body muscle activation, reduced musculoskeletal strain, and lower perceived workload during robotic procedures [31–33]. Although these benefits do not directly establish superior patient outcomes, they may support surgeon comfort and technical endurance, particularly during longer or more complex cases (Figure 4).

## Postoperative Outcomes

### Length of Stay and Recovery

Postoperative length of stay remains low with both robotic and laparoscopic cholecystectomy. In elective series, median hospital stay is approximately one day for both approaches [34,35]. Reported differences are generally small, although one single-surgeon study using a next-generation robotic platform found a 0.3-day reduction in length of stay with robotics (p=0.029) [36]. In acute or complex cases, findings are more variable. One multi-institutional acute-care analysis reported longer median postoperative stay with robotics (3 vs 2 days; p<0.001) [37], whereas a national propensity-matched cohort of more than 10,000 emergent cases found no significant difference after risk adjustment [8]. Collectively, available evidence indicates that robotic cholecystectomy neither prolongs recovery nor accelerates discharge in routine practice.

### Postoperative Pain and Analgesic Requirements

Several comparative studies report lower early postoperative pain after robotic cholecystectomy. In one cohort, 24-hour mean pain scores favored robotics (~1.8 vs 3.3 on a 10-point scale), accompanied by markedly reduced opioid use (0% vs 20%) [35]. Another prospective series reported lower pain scores in the robotic group on postoperative days 4 and 7 [34], and a single-surgeon study using a newer robotic platform similarly found reduced pain and opioid consumption with robotics [36]. These findings suggest a modest analgesic advantage, but they derive largely from nonrandomized settings with heterogeneous perioperative protocols and confirmation in higher-level studies is warranted.

### Wound Complications, Infection Rates, Readmission and Reoperation

Surgical site complications after minimally invasive cholecystectomy vary by clinical setting and patient population. In elective settings, a 2025 analysis of 116,703 patients using multivariate logistic regression with inverse probability treatment weighting found that robotic cholecystectomy was associated with decreased odds of readmission (OR 0.89, 95% CI 0.81-0.97, p=0.008) and hospital-acquired conditions (OR 0.71, 95% CI 0.60-0.83, p<0.001) compared with laparoscopic cholecystectomy [38].

By contrast, in a large acute care cohort, major complication rates were higher with robotics (8.4% vs 5.5%, p<0.001), although bile duct injury incidence was equivalent (~0.38% in both groups) [37]. This discrepancy may reflect higher acuity and greater procedural complexity among robotic cases, as many institutions deploy robotics preferentially for challenging anatomy or inflamed gallbladders.

Readmission and reoperation data are similarly mixed. Most large datasets, including the PINC AI national registry, found no difference in 30-day readmissions after adjustment for case mix [8]. Reported readmission rates across studies range from 1% to 3% in both groups. Reoperation remains rare (<1%) in modern series; however, a claims-based analysis of Medicare data found a modestly higher reoperation risk after robotics (relative risk ≈1.5 across risk strata) [26]. The authors suggested that this may relate to management of postoperative abscesses or bile leaks rather than technique-specific issues. Postoperative morbidity remains low and broadly comparable between robotic and laparoscopic cholecystectomy, although findings vary by patient population and clinical setting.

### Return to Activity

Return to activity is a relevant functional outcome, but data remain limited. Some prospective studies suggest slightly faster return to work or daily activity after robotic cholecystectomy, potentially paralleling reported reductions in early postoperative pain [34–36]. However, definitions of functional recovery vary across studies, and randomized data are lacking. Recovery appears at least equivalent between approaches, with any advantage of robotics remaining modest.

## Complex and High-Risk Scenarios

### Acute or Gangrenous Cholecystitis

Robotic cholecystectomy has been increasingly used in acute and severe cholecystitis, with mixed comparative findings. In a large national acute-care cohort, robotic and laparoscopic cholecystectomy had similar bile duct injury rates, but robotics was associated with higher unadjusted major complication rates and slightly longer median postoperative stay [37]. By contrast, a large real-world EHR analysis of emergent cholecystectomy found lower odds of conversion to open surgery with robotics, along with no difference in bile duct injury, surgical site infection, length of stay, mortality, or overall hospital cost after inverse probability treatment weighting [39]. Smaller single-center studies likewise support feasibility and low conversion in acute and gangrenous cases when robotics is performed in experienced programs [1].

In delayed inflammatory high-risk settings, robotic interval cholecystectomy after percutaneous cholecystostomy achieved morbidity and mortality comparable to laparoscopy without longer hospitalization or higher nonroutine discharge rates [40]. Robotic cholecystectomy therefore appears feasible in acute, gangrenous, and post-inflammatory disease, although any advantage remains selective and dependent on case mix and team experience.

### Obesity and Difficult Anatomy

Robotic systems are often selected for patients with obesity or altered biliary anatomy, where enhanced visualization, tremor filtration, and wristed articulation may mitigate technical difficulty. National datasets show that robotics is preferentially deployed in patients with BMI ≥40, particularly in emergency cases [8]. A 2025 single-institution analysis of 637 cholecystectomies in obese patients (BMI>30) found zero conversions to open surgery in robotic cases versus a 3.2% conversion rate in laparoscopic cases (p=0.038), along with slightly shorter mean hospital stays [41]. These findings suggest that robotics may help maintain minimally invasive access in technically challenging anatomy.

However, obesity itself remains a predictor of adverse outcomes irrespective of approach. A 2023 study of 617 robotic cholecystectomies reported a 9.1% complication rate, with class III obesity independently associated with postoperative complications (OR ~2.2) [22]. Robotics may facilitate safe completion of complex biliary dissections in obese patients, but extreme obesity continues to confer elevated baseline surgical risk even when robotic technology is employed.

### Reoperative or Post-Inflammatory Cases

Reoperative cholecystectomy after subtotal, aborted, or post-inflammatory procedures remains one of the most challenging operations in biliary surgery. Robotic assistance provides stable camera control and articulated instruments that can facilitate precise dissection through scarred or fibrotic planes. A 2025 multicenter cohort evaluating 44 robotic completion cholecystectomies after subtotal or aborted procedures demonstrated a 93% completion rate with only one open conversion (2.3%) and a 2.3% major complication rate, all in post-subtotal cases [42]. These results highlight the ability of robotics to safely navigate dense adhesions and support completion of cases that might otherwise require open conversion.

Smaller institutional series corroborate this favorable signal, showing equivalent or lower morbidity when robotics is used for post-inflammatory or reoperative gallbladders, often with adjunctive fluorescence imaging to delineate anatomy [43]. Robotic cholecystectomy provides a minimally invasive, controlled approach for complex reoperations, with favorable outcomes reported in hostile fields.

## Cost/Value Assessment

### Direct Operative Costs

Robotic cholecystectomy incurs substantially higher direct costs than laparoscopic surgery, largely because of expensive disposable instruments and system maintenance. Large health system analyses have quantified this difference. In one multihospital study (2017–2024, >14,000 cholecystectomies), the median disposable instrument cost was approximately $1,309 per robotic case versus $534 per laparoscopic case [15]. This roughly 2.5-fold per-case increase is consistent with findings reported across other institutions. Mixed-effects modeling further confirmed that, even after accounting for patient and surgeon factors, robotic cholecystectomy was on average $800 more expensive in supplies than laparoscopic cholecystectomy (p<0.001) [15]. Notably, the lowest-cost laparoscopic surgeons in that series averaged only $272 in instrument expenditures, illustrating the low baseline expense of conventional laparoscopy [15].

Comparative cohort studies consistently report higher direct costs with robotic than laparoscopic cholecystectomy. A propensity-matched analysis found that robotic cholecystectomy was associated with significantly higher operative and overall hospital costs compared with laparoscopic cholecystectomy, despite differences in selected perioperative outcomes [45]. Likewise, a single-center retrospective analysis of elective cases demonstrated higher total hospital costs for robotic procedures in Europe (€2,088 vs €1,726), even in the setting of equivalent operative times, reflecting increased procedural and system-related expenditures [21].

Beyond per-case disposable costs, robotic platforms also carry substantial capital and maintenance expenses. Acquisition costs frequently exceed several million dollars. Annual service contracts further increase expenses and, when amortized under typical utilization assumptions, add approximately $1,000 or more per cholecystectomy [46]. For example, fixed system costs for a single-site robotic procedure have been estimated at $3,568 per case, including approximately $1,038 attributable to the robotic platform and $663 for service contracts [46]. These factors more than double the operative hardware expense for cholecystectomy. Higher direct costs reflect proprietary robotic instruments, including staplers and energy devices, as well as single-use drapes and platform-specific consumables required for each case.

## Indirect Costs (Time and Throughput)

Beyond instruments, robotic surgery may introduce indirect costs related to operative time, operating-room throughput, and training during program implementation. Early adoption studies of robotic cholecystectomy and other benign abdominal procedures have reported longer room times related to docking, setup, and workflow unfamiliarity during initial cases [22,47,48]. Time-motion and implementation analyses further show that docking and setup contribute meaningfully to total room time early in adoption, with substantial interinstitutional variability depending on team experience and operating-room configuration [48,49].

Learning-curve analyses consistently show that operative efficiency improves with experience. Cumulative sum and institutional rollout studies indicate that robotic console and total operative times decline over the first several dozen cases, approaching laparoscopic benchmarks once surgeons and teams move beyond the early learning phase [22,48]. Program-level implementation studies further suggest that this stabilization reflects surgeon experience as well as improvements in team coordination, troubleshooting, and standardized room setup [49,50].

Even modest increases in operative duration can carry economic consequences in high-volume operating rooms. Health-systems and implementation studies emphasize that operating-room time is a major driver of hospital cost, so small per-case increases in room time may reduce daily throughput or increase staffing and anesthesia expenditures when scaled across large procedural volumes [50,51]. In addition, adoption of robotic platforms requires institutional investment in team training, simulation, and early proctoring, which represent indirect costs during program initiation [49,50].

In acute-care and nonelective settings, some observational studies have reported slightly longer postoperative stays with robotic cholecystectomy, potentially reflecting greater case complexity, drain utilization, or selection factors during early adoption [37]. In elective practice, however, length of stay is typically equivalent between robotic and laparoscopic approaches, with most patients discharged within 24 hours regardless of platform [37]. High-volume centers and mature programs also report no significant differences in mean operative time once surgeons and teams are beyond the learning curve [20,37]. Indirect costs related to time and training are concentrated in the early implementation phase and may diminish substantially with experience and optimized workflows.

### Cost-Effectiveness Analyses

Multiple recent studies have assessed whether the improved technical capabilities of robotics justify the higher associated costs. In routine cholecystectomy, current cost-effectiveness analyses continue to favor laparoscopy. A 2023 decision-tree cost-utility analysis compared robotic and laparoscopic cholecystectomy using published complication rates and quality-of-life data [52]. Laparoscopic cholecystectomy was calculated to produce 0.9722 quality-adjusted life years (QALYs) at a cost of $9,370 per patient [52]. Robotic cholecystectomy yielded a nearly identical QALY (0.9739, an incremental gain of 0.0017 QALY) at an additional cost of $3,014 [52]. This resulted in an incremental cost-effectiveness ratio (ICER) of roughly $1.8 million per QALY gained with robotics [52]. Such an ICER greatly exceeds commonly accepted willingness-to-pay thresholds, indicating that laparoscopy remains the economically dominant strategy [52]. Similarly, a 2024 systematic review and meta-analysis encompassing >22,000 patients found no significant clinical advantages of robotic over laparoscopic cholecystectomy, while consistently reporting higher costs with the robotic approach [14,53]. The authors concluded that widespread robotic cholecystectomy is not justified without evidence of superior outcomes, given the cost disparity [14,53]. These analyses indicate that current-generation robotic cholecystectomy is not cost-effective in uncomplicated cases.

### Economic Threshold for Parity

For robotic cholecystectomy to approach cost parity with laparoscopy, either platform-related costs would need to decline substantially, or the robotic approach would need to deliver clinically meaningful benefits that offset its added expense. One potential offset is avoidance of open conversion and its associated downstream morbidity and cost. However, although conversion may carry an estimated downstream cost of roughly $10,000 to $20,000, the cost per conversion averted with robotics has been estimated at approximately $93,000 [15]. This does not represent a cost-effective trade-off in routine practice. More broadly, a 2023 national analysis found that the cost gap between robotic and laparoscopic abdominal surgery persisted and widened from 2012 to 2019 despite only modest outcome improvements, including an approximately 2% reduction in complications and less than a 1-day decrease in length of stay [54]. Under current practice conditions, cost parity appears unlikely in routine elective cholecystectomy and would be more plausible only if robotic costs decline or if selective use in higher-risk cases yields clearer clinical benefit.

## Patient-Reported and Functional Outcomes

### Cosmetic Satisfaction

Cosmetic outcome is often cited as a potential advantage of robotic cholecystectomy, particularly in single-incision or reduced-port approaches [16]. In prospective comparative data, single-incision robotic cholecystectomy has been associated with higher patient satisfaction with scar appearance and body image than conventional multiport laparoscopy [55]. However, when standard multiport techniques are used for both robotic and laparoscopic cholecystectomy, comparative studies have not demonstrated consistent differences in cosmetic or quality-of-life outcomes [56]. Cosmetic benefits therefore appear most relevant in selected reduced-port applications and should be weighed against their added technical complexity and resource use [57].

### Quality of Life and Recovery

A central goal of minimally invasive cholecystectomy is to facilitate rapid recovery while preserving postoperative quality of life (QoL). Patient-reported outcome studies have evaluated robotic and laparoscopic cholecystectomy with respect to postoperative pain, functional recovery, and overall well-being. Within cholecystectomy-specific cohorts, short-term recovery following robotic cholecystectomy appears at least equivalent to laparoscopy. Several non-randomized studies have reported modest reductions in early postoperative pain and analgesic requirements with robotic approaches, particularly during the first postoperative week [58]. This may reflect technical features of the robotic platform, such as wristed instrumentation and reduced abdominal wall torque, although these explanations remain speculative and untested.

Observational data in this domain are heterogeneous. Some single-center and retrospective comparisons have reported similar postoperative pain trajectories and recovery timelines between robotic and laparoscopic approaches, despite differences in operative platform and technique [59]. Moreover, the observational nature of many early comparative studies introduces the potential for selection bias, as patient characteristics and surgeon preference may influence both procedural choice and reported outcomes. Consistent with this variability, other comparative series, including single-surgeon analyses of single-incision robotic versus conventional laparoscopic cholecystectomy, have demonstrated no significant differences in postoperative pain or short-term recovery metrics between approaches [60].

In contrast to observational findings, higher-level evidence has not demonstrated consistent or sustained advantages for either approach with respect to patient-reported pain or functional recovery. Systematic reviews of randomized controlled trials have shown no statistically significant differences in early postoperative pain between robotic and laparoscopic cholecystectomy, although isolated trials have reported modest, short-lived trends toward reduced analgesic use with robotic techniques that are not uniformly reproduced across studies [56]. Importantly, longer-term patient-reported outcomes appear comparable between approaches. In a single-institution study assessing validated QoL and pain instruments between two and seven years after surgery, no meaningful differences were observed in overall QoL or pain intensity between robotic and laparoscopic cholecystectomy, with only minor variations in select pain subdomains [61]. Similarly, standardized QoL measures such as the SF-36 and EQ-5D have not demonstrated sustained differences beyond the early postoperative period, reflecting the generally low morbidity and rapid functional recovery associated with cholecystectomy regardless of operative platform [61,62].

The available evidence indicates that patient-reported outcomes following robotic cholecystectomy are non-inferior to those achieved with laparoscopy [56,63]. While selected studies suggest potential benefits in immediate postoperative comfort with robotic approaches, these effects are modest, context-dependent, and not consistently supported by randomized data [56,60,64]. From a patient-centered perspective, both techniques reliably achieve rapid recovery and favorable quality-of-life outcomes when performed in appropriate clinical settings.

### Patient Preferences and Satisfaction

Robotic surgery appears to influence patient perception and procedural preference, with some patients viewing robotic cholecystectomy as a more advanced or precise approach [11]. Satisfaction among patients undergoing robotic cholecystectomy is generally high [57], but this is not unique to robotics, as laparoscopic cholecystectomy is also associated with consistently favorable patient experience [65]. Available evidence suggests that differences in satisfaction between approaches are modest and are shaped by expectations, communication, and early postoperative experience rather than by durable differences in quality of life or functional recovery [11,58]. Cosmetic expectations may also contribute to perceived benefit in selected patients [50]. Clear preoperative counseling remains important to align expectations and clarify the surgeon’s role in robotic procedures [63].

## Discussion

### Interpretation of Comparative Safety and Cost Findings

The contemporary literature comparing robotic and laparoscopic cholecystectomy remains heterogeneous, particularly with respect to safety [11,12]. Population-based analyses have raised concern regarding bile duct injury and overall morbidity with robotic cholecystectomy [11], whereas other national and institutional studies have reported equivalent or, in selected settings, improved outcomes such as lower conversion rates [7,17]. These discrepancies likely reflect differences in case selection, adoption phase, and institutional experience rather than the operative platform alone [2,22]. Cost findings are more consistent. Robotic cholecystectomy is associated with higher direct operative costs, and although technical advantages may be relevant in complex cases, these benefits have not translated into sufficient routine postoperative improvement to offset the added expense in standard gallbladder disease [15,52] (Figure 2).

## Methodological Constraints in the Existing Evidence Base

The mixed conclusions in the literature are largely attributable to limitations inherent to the current body of evidence. Most comparative studies are retrospective and observational, relying on administrative or registry data that lack granular detail regarding disease severity, intraoperative decision-making, and surgeon experience. Selection bias is pervasive, as robotic cholecystectomy is often preferentially applied to patients with obesity, prior inflammation, or complex anatomy, particularly in acute care settings [8]. Even with propensity matching or multivariable adjustment, residual confounding remains likely, particularly related to procedural volume. Large nationwide registry data demonstrate that low surgeon and hospital cholecystectomy volumes are independently associated with higher conversion rates, bile duct injury, and mortality in acute cases, irrespective of surgical approach, complicating attribution of adverse outcomes to operative platform alone [66]. Additionally, there is substantial heterogeneity in operative technique, including differences in port number, single-site versus multiport approaches, and adjunctive technologies such as fluorescence imaging [16]. These factors confound comparisons between platforms and complicate attribution of outcomes to the robotic system itself. Finally, the absence of randomized controlled trials limits causal inference. As a result, observed differences in safety or cost should be interpreted as associations within specific clinical contexts, rather than definitive evidence of platform superiority or inferiority.

## Learning Curve, Technology Maturity, and Institutional Effects

Learning curve effects play a central role in reconciling conflicting safety data [22]. Early robotic cholecystectomy experience was marked by longer operative times and higher complication rates, whereas contemporary series demonstrate improved efficiency and safety as surgeon and team experience accrue. Early population-level experience supports this interpretation. In a statewide analysis of New York cholecystectomies performed between 2009 and 2017, robot-assisted cholecystectomy was associated with higher rates of conversion to open surgery, bile duct injury, readmission, and longer length of stay compared with laparoscopy [67]. Institutional factors, including case volume, standardized workflows, and structured proctoring, appear to exert a greater influence on outcomes than the choice of minimally invasive platform alone. High-volume centers with formal robotic training pathways consistently report low conversion rates and acceptable complication profiles, suggesting that mentorship and system-level support mitigate early adoption risks [43]. Similarly, a Veterans Affairs system that transitioned fully to robotics reduced conversion rates from 14.6% to 0% without increased complications or readmissions [44]. Technological maturation has also contributed to improved performance, with advances in visualization, ergonomics, and instrument design facilitating more precise dissection in difficult cases. Nonetheless, these benefits are realized primarily in environments where robotic surgery is integrated thoughtfully rather than deployed indiscriminately. Early prospective evaluation of novel robotic platforms under structured innovation frameworks further illustrates this principle. In an IDEAL-D Stage 2b prospective clinical study of robot-assisted cholecystectomy using a next-generation system, procedures were completed with acceptable conversion and complication rates under independent adverse-event adjudication, supporting cautious, staged implementation rather than indiscriminate adoption [68].

## Implications for General Surgery Practice and Training

The expanding role of robotics in cholecystectomy has important implications for general surgery training. Robotic cholecystectomy is increasingly used as an entry procedure for resident console training and early robotic skill development [43]. Available evidence suggests that, within structured programs, trainees can safely participate in robotic cholecystectomy without increased complication rates. However, variability in resident autonomy, access to dual-console systems, and formal curricula remains substantial. At the same time, concerns persist that increasing robotic exposure may detract from the acquisition of core laparoscopic skills, particularly as conversion to open surgery becomes less common [30]. These findings underscore the need for balanced training models that ensure competency across laparoscopic, robotic, and open techniques, with clearly defined milestones for robotic proficiency rather than case-count–based exposure alone.

## Resource Allocation and Selective Adoption

From a health systems perspective, the routine use of robotic cholecystectomy for uncomplicated gallbladder disease is difficult to justify given its higher cost and lack of consistent outcome superiority [52]. The literature instead supports a selective adoption strategy, reserving robotic cholecystectomy for patients in whom technical advantages may meaningfully reduce conversion risk or operative difficulty, such as those with obesity, dense inflammation, or prior biliary intervention [41]. A similar context-specific signal has been observed in patients with advanced liver disease. In a NSQIP analysis stratified by MELD score, robotic cholecystectomy was associated with lower conversion rates and shorter length of stay compared with laparoscopy, while maintaining comparable perioperative mortality in higher-MELD patients, suggesting a potential role for robotics in carefully selected high-risk populations [69]. In these contexts, preventing conversion or major complications may partially offset the higher procedural cost. Hospitals and surgical programs should therefore view robotic cholecystectomy as a complementary tool rather than a universal replacement for laparoscopy, particularly in settings where case complexity and clinical objectives justify its use. Ongoing evaluation of outcomes and costs, particularly as new robotic platforms enter the market, will be essential to refining this selective framework. Future resource-allocation decisions may also need to incorporate environmental sustainability, as life-cycle modeling of laparoscopic cholecystectomy suggests that reusable instruments, anesthetic optimization, and reduced disposable components can lower procedural carbon emissions while remaining cost-neutral or cost-saving [70].

## Conclusion

Robotic cholecystectomy has not demonstrated consistent superiority over laparoscopic cholecystectomy for routine gallbladder disease. Contemporary evidence supports comparable perioperative safety in many settings and suggests potential advantages in selected technically complex cases, particularly with respect to conversion to open surgery. However, higher direct and indirect costs remain a major limitation, and current economic data do not support broad routine adoption. The most defensible role for robotic cholecystectomy is therefore selective use in patients or operative settings where its technical advantages may reasonably offset added cost and resource utilization. The next phase of evidence should move beyond broad platform comparisons toward prospective, severity-adjusted studies that incorporate surgeon experience, patient-centered recovery, and full cost accounting to determine where robotic cholecystectomy provides measurable value.

## Key Points

- Robotic cholecystectomy has expanded rapidly in clinical practice, but consistent superiority over laparoscopic cholecystectomy has not been demonstrated for routine gallbladder disease
- Reduced conversion to open surgery is one of the most recurrent favorable findings associated with robotic cholecystectomy, particularly in technically complex cases
- Safety findings remain mixed, with population-level studies raising concern regarding bile duct injury and contemporary institutional, registry, and nonelective studies reporting low or comparable bile duct injury rates
- Postoperative recovery, length of stay, readmission, reoperation, quality of life, and patient satisfaction are generally similar between robotic and laparoscopic approaches
- Higher direct and indirect costs remain a major limitation of robotic cholecystectomy, and current economic evidence does not support broad routine adoption for uncomplicated disease
- Selective use may be most appropriate when case complexity, operative difficulty, or conversion risk provides a plausible rationale for the added cost and resource utilization

## References

[1] Rhodes MA, Otero J, Rochester SN, Comparative Analysis of Laparoscopic and Robotic Cholecystectomy: A Multihospital Retrospective Study. JSLS 29 (2025): e2024.00068. 10.4293/JSLS.2024.00068 PMC11967723 40182833

[2] Sheetz KH, Claflin J, Dimick JB. Trends in the Adoption of Robotic Surgery for Common Surgical Procedures. JAMA Netw Open 3 (2020): e1918911. 10.1001/jamanetworkopen.2019.18911 PMC6991252 31922557

[3] Aabedi A, Mashiach D, Fraix MP, Most Effective Interventions for Improving Upper Extremity Function in Patients with Hemiparesis. Cardiol Cardiovasc Med 9 (2026): 504–511. 10.26502/fccm.92920475 PMC13108678 42037657

[4] Azarraga RB, Jackson MC, Fraix MP, Innovation, Adaptation, and Human Dignity in Assistive Robotics in Amyotrophic Lateral Sclerosis: A Rehabilitation Medicine Perspective. J Biotechnol Biomed 9 (2026): 28–39. 10.26502/jbb.2642-91280207 PMC12970945 41810116

[5] Postajian A, Rostomian E, Abdou A, Management of Venous Thromboembolism After Hip and Knee Arthroplasty. J Orthop Sports Med 7 (2025): 311–327. 10.26502/josm.511500210 PMC12376865 40855902

[6] Rostomian E, Ghookas K, Postajian A, Innovative Approaches for the Treatment of Spinal Disorders: A Comprehensive Review. J Orthop Sports Med 7 (2025): 144–161. 10.26502/josm.511500190 PMC12040341 40303932

[7] Parvizi D, Han B, Allahverdian A, Merits and Limitations of Robotic-assisted Surgery in Improving Precision, Accuracy, and Patient Outcomes in Orthopedic Procedures. J Orthop Sports Med 7 (2025): 569–578. 10.26502/josm.511500243 PMC12948186 41768484

[8] Campbell S, Lee SH, Liu Y, A retrospective study of laparoscopic, robotic-assisted, and open emergent/urgent cholecystectomy based on the PINC AI Healthcare Database 2017–2020. World J Emerg Surg 18 (2023): 55. 10.1186/s13017-023-00521-8 PMC10687827 38037087

[9] Kawka M, Jawad ZAR, Hakim D, Robotic versus laparoscopic cholecystectomy for difficult gallbladders: an observational study of tertiary centre cases. Surg Endosc 39 (2025): 2958–2963. 10.1007/s00464-025-11586-8 PMC12041051 40113617

[10] Brnawi H, Alenzi FKA, Aljohani AJS, Robotic versus Laparoscopic Cholecystectomy in Cholecystitis: Precision Meets Tradition – A Review of Surgical Techniques: A Meta-analysis. Journal of Advanced Trends in Medical Research 2 (2025): 145–160.

[11] Kalata S, Thumma JR, Norton EC, Comparative Safety of Robotic-Assisted vs Laparoscopic Cholecystectomy. JAMA Surg 158 (2023): 1303. 10.1001/jamasurg.2023.4389 PMC10512167 37728932

[12] Maegawa FB, Stetler J, Patel D, Robotic compared with laparoscopic cholecystectomy: A National Surgical Quality Improvement Program comparative analysis. Surgery 178 (2025): 108772. 10.1016/j.surg.2024.08.006 39277483

[13] Tao Z, Emuakhagbon VS, Pham T, Outcomes of robotic and laparoscopic cholecystectomy for benign gallbladder disease in Veteran patients. J Robotic Surg 15 (2021): 849–857. 10.1007/s11701-020-01183-3 33400103

[14] Singh A, Kaur M, Swaminathan C, Laparoscopic versus robotic cholecystectomy: a systematic review with meta-analysis to differentiate between postoperative outcomes and cost-effectiveness. Transl Gastroenterol Hepatol 9 (2024): 3. 10.21037/tgh-23-56 PMC10838610 38317744

[15] Dallal RM, Araya S, Sadeh JI, Impact of the robotic platform and surgeon variation on cholecystectomy disposable costs—More is not better. Surgery 183 (2025): 109332. 10.1016/j.surg.2025.109332 40113517

[16] Jeong Jang E, Kang SH, Kim KW. Early Outcomes of Robotic Single Site Cholecystectomy Using the DaVinci Xi^®^ System. JSLS 25 (2021): e2020.00082. 10.4293/JSLS.2020.00082 PMC7881280 33628004

[17] Khanna S, Barua A. Robotic assisted cholecystectomy – A retrospective cohort study of experience of 106 first robotic cholecystectomies in versius robotic platform. International Journal of Surgery Open 47 (2022): 100554.

[18] Kim WJ, Choi SB, Kim WB. Feasibility and Efficacy of Single-Port Robotic Cholecystectomy Using the da Vinci SP^®^ Platform. JSLS 26 (2022): e2021.00091. 10.4293/JSLS.2021.00091 PMC9205460 35815324

[19] Mintz Y, Elazary R, Helou B, A simple technique to improve docking time in robotic surgery. J Robotic Surg 19 (2024): 14. 10.1007/s11701-024-02179-z PMC11609118 39616597

[20] Kwon W, Jang JY, Jeong CW, Cholecystectomy with the HugoTM robotic-assisted surgery system: the first general surgery clinical study in Korea. Surg Endosc 39 (2025): 171–179. 10.1007/s00464-024-11334-4 PMC11666616 39466427

[21] Gantschnigg A, Koch OO, Singhartinger F, Short-term outcomes and costs analysis of robotic-assisted versus laparoscopic cholecystectomy—a retrospective single-center analysis. Langenbecks Arch Surg 408 (2023): 299. 10.1007/s00423-023-03037-6 PMC10409838 37552295

[22] Kudsi OY, Kaoukabani G, Friedman A, Learning Curve of Single-site Robotic Cholecystectomy: A Cumulative Sum Analysis. Surgical Laparoscopy, Endoscopy & Percutaneous Techniques 33 (2023): 310–316. 10.1097/SLE.0000000000001178 37172003

[23] Villani V, Kao LS, Fong Y. The Difficult Cholecystectomy. JAMA Surg (2025). 10.1001/jamasurg.2025.4199 41091499

[24] Shen A, Barmparas G, Melo N, Incorporating Robotic Cholecystectomy in an Acute Care Surgery Practice Model is Feasible. The American SurgeonTM 90 (2024): 2457–2462. 10.1177/00031348241248816 38654460

[25] Camarotti TDAF Lenzi MC, Cardoso JHCO, Clinical outcomes of robotic-assisted versus laparoscopic cholecystectomy in nonelective procedures: A systematic review and meta-analysis. J Trauma Acute Care Surg (2025). 10.1097/TA.0000000000004851 41417656

[26] Mullens CL, Sheskey S, Thumma JR, Patient Complexity and Bile Duct Injury After Robotic-Assisted vs Laparoscopic Cholecystectomy. JAMA Netw Open 8 (2025): e251705. 10.1001/jamanetworkopen.2025.1705 PMC11937934 40131276

[27] Dip F, Lo Menzo E, White KP, Does near-infrared fluorescent cholangiography with indocyanine green reduce bile duct injuries and conversions to open surgery during laparoscopic or robotic cholecystectomy? — A meta-analysis. Surgery 169 (2021): 859–867. 10.1016/j.surg.2020.12.008 33478756

[28] Pimentel T, Queiroz I, Gallo Ruelas M, Indocyanine green fluorescent cholangiography in laparoscopic cholecystectomy: A systematic review and meta-analysis with trial sequential analysis of randomized controlled trials. Surgery 181 (2025): 109149. 10.1016/j.surg.2025.109149 39891966

[29] Broderick RC, Lee AM, Cheverie JN, Fluorescent cholangiography significantly improves patient outcomes for laparoscopic cholecystectomy. Surg Endosc 35 (2021): 5729–5739. 10.1007/s00464-020-08045-x 33052527

[30] Urbach DR. Robotic-Assisted Cholecystectomy—for Whom? JAMA Netw Open 8 (2025): e251711. 10.1001/jamanetworkopen.2025.1711 40131279

[31] Zihni AM, Ohu I, Cavallo JA, Ergonomic analysis of robot-assisted and traditional laparoscopic procedures. Surg Endosc 28 (2014): 3379–3384. 10.1007/s00464-014-3604-9 24928233

[32] Cooper H, Lau HM, Mohan H. A systematic review of ergonomic and muscular strain in surgeons comparing robotic to laparoscopic approaches. J Robotic Surg 19 (2025): 252. 10.1007/s11701-025-02401-6 PMC12126336 40448883

[33] Wee IJY, Kuo L, Ngu JC. A systematic review of the true benefit of robotic surgery: Ergonomics. Robotics Computer Surgery 16 (2020): e2113. 10.1002/rcs.2113 32304167

[34] Wadhawan R, Galhotra A, Veetil DK, Perioperative and Patient-Reported Clinical Outcomes of Robotic Versus Laparoscopic Cholecystectomy. JSLS 28 (2024): e2024.00051. 10.4293/JSLS.2024.00051 PMC11935298 40134931

[35] Ray U, Dhar R. A Retrospective Analysis of Short-Term Outcomes of Robotic and Laparoscopic Cholecystectomy: An Indian Tertiary Care Comparative Experience. Cureus (2024). 10.7759/cureus.69295 PMC11470972 39398781

[36] Oner M. Preliminary comparative outcomes of Versius Robotic System-assisted cholecystectomy and laparoscopic cholecystectomy for benign gall bladder disease: Retrospective single-centre, single-surgeon analysis. Journal of Minimal Access Surgery 21 (2025): 378–384. 10.4103/jmas.jmas_13_24 PMC12585146 40136019

[37] Woldehana NA, Jung A, Parker BC, Clinical Outcomes of Laparoscopic vs Robotic-Assisted Cholecystectomy in Acute Care Surgery. JAMA Surg 160 (2025): 755. 10.1001/jamasurg.2025.1291 PMC12096326 40397430

[38] Abou Assali M, Li Y, Bossie H, Robotic Care Outcomes Project (ROBOCOP) for elective cholecystectomy. Surg Endosc 39 (2025): 7262–7271. 10.1007/s00464-025-12109-1 PMC12618350 40858944

[39] Greenberg S, Abou Assali M, Li Y, ROBOtic Care Outcomes Project for acute gallbladder pathology. J Trauma Acute Care Surg 96 (2024): 971–979. 10.1097/TA.0000000000004240 38189678

[40] Nzenwa IC, Sanyal R, Arda Y, Robot-assisted Interval Cholecystectomy is not Inferior to Laparoscopic Interval Cholecystectomy in Advanced Cholecystitis. Journal of Surgical Research 315 (2025): 313–323. 10.1016/j.jss.2025.09.040 41072094

[41] Sebastian R, Zevallos A, Montenegro D, Robotic cholecystectomy reduces the conversion rate in patients with obesity. The Surgeon (2025). 10.1016/j.surge.2025.07.004 40653401

[42] Stefanova I, Callahan R, Giriradder VB, The final cut: a multi-centre cohort study evaluating outcomes of robotic completion cholecystectomy. Surg Endosc (2025). 10.1007/s00464-025-12044-1 PMC12618356 40877634

[43] Stefanova I, Alkhatib O, Sheel A, Safety of robotic cholecystectomy as index training procedure: the UK experience. Surg Endosc 38 (2024): 4880–4886. 10.1007/s00464-024-11006-3 38955837

[44] Huy TC, Fitzsimmons K, Park J, The robotic era: 11-year retrospective study of cholecystectomies at a veterans affairs hospital. Surg Endosc (2025). 10.1007/s00464-025-12185-3 PMC12618365 40905966

[45] Kane WJ, Charles EJ, Mehaffey JH, Robotic compared with laparoscopic cholecystectomy: A propensity matched analysis. Surgery 167 (2020): 432–435. 10.1016/j.surg.2019.07.020 PMC6980975 31492434

[46] Rudiman R, Hanafi RV, Almawijaya A. Single-site robotic cholecystectomy versus single-incision laparoscopic cholecystectomy: A systematic review and meta-analysis. Annals of Gastroent Surgery 7 (2023): 709–718. 10.1002/ags3.12688 PMC10472369 37663974

[47] Sheetz KH, Thumma JR, Kalata S, Learning Curve for Robotic-Assisted Cholecystectomy. JAMA Surg 159 (2024): 833. 10.1001/jamasurg.2024.1221 PMC11112490 38776095

[48] Seeger N, Mignot H, Matthaei H, Robotic-assisted cholecystectomy with DEXTER^®^: the first prospective multicenter study. Surg Endosc 39 (2025): 8254–8262. 10.1007/s00464-025-12174-6 PMC12708809 40973903

[49] Hotz A, Seeger N, Gantner L, Implementation of a Robotic Surgical Program With the Dexter Robotic Surgery System: Initial Experiences in Cholecystectomy. World J Surg 49 (2025): 1221–1227. 10.1002/wjs.12531 PMC12058442 40122784

[50] Klein J, Lemma M, Prabhakaran K, Robotic versus Laparoscopic Emergency and Acute Care Surgery: Redefining Novelty (RLEARN): feasibility and benefit of robotic cholecystectomy for acute cholecystitis at a level 1 trauma center. Trauma Surg Acute Care Open 9 (2024): e001522. 10.1136/tsaco-2024-001522 PMC11683923 39737144

[51] Childers CP, Maggard-Gibbons M. Understanding Costs of Care in the Operating Room. JAMA Surg 153 (2018): e176233. 10.1001/jamasurg.2017.6233 PMC5875376 29490366

[52] Singh A, Panse NS, Prasath V, Cost-effectiveness analysis of robotic cholecystectomy in the treatment of benign gallbladder disease. Surgery 173 (2023): 1323–1328. 10.1016/j.surg.2023.01.017 36914510

[53] Tawde P, John N, Farah S, Comparison of da Vinci Robotic Cholecystectomy and Laparoscopic Cholecystectomy: A Systematic Review and Meta-Analysis of Postoperative Outcomes and Cost-Effectiveness. Cureus (2024). 10.7759/cureus.73767 PMC11650005 39691126

[54] Ng AP, Sanaiha Y, Bakhtiyar SS, National analysis of cost disparities in robotic-assisted versus laparoscopic abdominal operations. Surgery 173 (2023): 1340–1345. 10.1016/j.surg.2023.02.016 36959072

[55] Kudsi OY, Castellanos A, Kaza S, Cosmesis, patient satisfaction, and quality of life after da Vinci Single-Site cholecystectomy and multiport laparoscopic cholecystectomy: short-term results from a prospective, multicenter, randomized, controlled trial. Surg Endosc 31 (2017): 3242–3250. 10.1007/s00464-016-5353-4 27864724

[56] Kawka M, Fong Y, Gall TMH. Laparoscopic versus robotic abdominal and pelvic surgery: a systematic review of randomised controlled trials. Surg Endosc 37 (2023): 6672–6681. 10.1007/s00464-023-10275-8 PMC10462573 37442833

[57] Spurzem GJ, Jimenez J, Paravic N, Solo surgeon ambulatory magnetic-assisted robotic surgery (MARS): initial 51 cases with high patient satisfaction. Surg Endosc 39 (2025): 4463–4469. 10.1007/s00464-025-11879-y PMC12222352 40500543

[58] Lee SJ, Moon JI, Choi IS. Robotic single-site cholecystectomy is better in reducing postoperative pain than single-incision and conventional multiport laparoscopic cholecystectomy. Surg Endosc 37 (2023): 3548–3556. 10.1007/s00464-022-09846-y 36604338

[59] Rifai AO, Rembetski EM, Stutts LC, Retrospective analysis of operative time and time to discharge for laparoscopic vs robotic approaches to appendectomy and cholecystectomy. J Robotic Surg 17 (2023): 2187–2193. 10.1007/s11701-023-01632-9 PMC10492745 37271758

[60] Lee SR, Kim HO, Shin JH. Clinical outcomes of single-incision robotic cholecystectomy versus conventional 3-port laparoscopic cholecystectomy. CJS 62 (2019): 52–56. 10.1503/cjs.000118 PMC6351249 30693746

[61] Mudgway R, Tran Z, Quispe Espíritu JC, A Medium-Term Comparison of Quality of Life and Pain After Robotic or Laparoscopic Cholecystectomy. Journal of Surgical Research 295 (2024): 47–52. 10.1016/j.jss.2023.08.031 37988906

[62] Repo A, Eskelinen M, Saimanen I, Patient-reported Outcome Measure (PROM) Rand-36-item Health Survey for Gallstone Disease Patients Five Years Following Surgery: A Prospective Randomized Study. In Vivo 38 (2024): 1213–1219. 10.21873/invivo.13557 PMC11059901 38688655

[63] Tang K, Zhou W, Zhou Y, Robotic-assisted versus conventional/single-incision laparoscopic cholecystectomy for benign gallbladder disease: A systematic review and meta-analysis. Medicine 104 (2025): e42493. 10.1097/MD.0000000000042493 PMC12114010 40419930

[64] Pietrabissa A, Pugliese L, Vinci A, Short-term outcomes of single-site robotic cholecystectomy versus four-port laparoscopic cholecystectomy: a prospective, randomized, double-blind trial. Surg Endosc 30 (2016): 3089–3097. 10.1007/s00464-015-4601-3 26497946

[65] McLean KA, Sheng Z, O’Neill S, The Influence of Clinical and Patient-Reported Outcomes on Post-surgery Satisfaction in Cholecystectomy Patients. World J Surg 41 (2017): 1752–1761. 10.1007/s00268-017-3917-7 PMC5486820 28280919

[66] Blohm M, Sandblom G, Enochsson L, Relationship between surgical volume and outcomes in elective and acute cholecystectomy: nationwide, observational study. British Journal of Surgery 110 (2023): 353–361. 10.1093/bjs/znac415 PMC10364541 36422988

[67] Hoffman AB, Myneni AA, Towle-Miller LM, The Early (2009–2017) Experience With Robot-assisted Cholecystectomy in New York State. Annals of Surgery 274 (2021): e245–e252. 10.1097/SLA.0000000000004932 34397456

[68] Kelkar DS, Kurlekar U, Stevens L, An Early Prospective Clinical Study to Evaluate the Safety and Performance of the Versius Surgical System in Robot-Assisted Cholecystectomy. Annals of Surgery 277 (2023): 9–17. 10.1097/SLA.0000000000005410 PMC9762713 35170538

[69] Aziz H, Zeeshan M, Kaur N, A Potential Role for Robotic Cholecystectomy in Patients with Advanced Liver Disease: Analysis of the NSQIP Database. Am Surg 86 (2020): 341–345. 32391758

[70] Bluiminck S, Eussen MMM, Kooistra EJ, Reducing the carbon footprint and costs of laparoscopic cholecystectomy: a modelling study on interventions for sustainable surgery. Surg Endosc (2025). 10.1007/s00464-025-12471-0 41419698

